# The Epidemiology of Myeloproliferative Neoplasms in New Zealand between 2010 and 2017: Insights from the New Zealand Cancer Registry

**DOI:** 10.3390/curroncol28020146

**Published:** 2021-04-18

**Authors:** Chris Varghese, Tracey Immanuel, Anna Ruskova, Edward Theakston, Maggie L. Kalev-Zylinska

**Affiliations:** 1 Blood and Cancer Biology Laboratory, Department of Molecular Medicine & Pathology, School of Medical Sciences, The University of Auckland, Auckland 1023, New Zealand; cvar706@aucklanduni.ac.nz (C.V.); tracey.immanuel@auckland.ac.nz (T.I.); 2 Department of Pathology and Laboratory Medicine, Auckland City Hospital, Auckland 1023, New Zealand; aruskova@adhb.govt.nz (A.R.); edwardt@adhb.govt.nz (E.T.)

**Keywords:** myeloproliferative neoplasms, polycythaemia vera, essential thrombocythaemia, primary myelofibrosis, epidemiology, incidence rates, capture rates, survival, ethnic disparity, cancer

## Abstract

Background: There is a paucity of data on ethnic disparities in patients with the classical Philadelphia chromosome-negative myeloproliferative neoplasms (MPNs): polycythaemia vera (PV), essential thrombocythaemia (ET) and primary myelofibrosis (PMF). Methods: This study analysed the demographic data for PV, ET and PMF collected by the New Zealand Cancer Registry (NZCR) between 2010 and 2017. Results: We found that the NZCR capture rates were lower than average international incidence rates for PV and ET, but higher for PMF (0.76, 0.99 and 0.82 per 100,000, respectively). PV patients were older and had worse outcomes than expected, which suggests these patients were reported to the registry at an advanced stage of their disease. Polynesian patients with all MPN subtypes, PV, ET and PMF, were younger than their European counterparts both at the time of diagnosis and death (*p* < 0.001). Male gender was an independent risk factor for mortality from PV and PMF (hazard ratios (HR) of 1.43 and 1.81, respectively; *p* < 0.05), and Māori ethnicity was an independent risk factor for mortality from PMF (HR: 2.94; *p* = 0.006). Conclusions: New Zealand Polynesian patients may have increased genetic predisposition to MPN, thus we advocate for modern genetic testing in this ethnic group to identify the cause. Further work is also required to identify modifiable risk factors for mortality in MPN, in particular those associated with male gender and Māori ethnicity; the results may benefit all patients with MPN.

## 1. Introduction

Classical Philadelphia chromosome-negative (Ph-negative) myeloproliferative neoplasms (MPNs) include the clinical entities of polycythaemia vera (PV), essential thrombocythaemia (ET) and primary myelofibrosis (PMF). MPNs are associated with a significant reduction in patients’ quality of life and an increased mortality risk [1,2,3]. Significant efforts are ongoing to develop therapies that halt MPN progression; epidemiological data are crucial to guide such efforts.

MPNs are not uncommon. A systematic review reported the combined international incidence rates to be 0.84, 1.03, and 0.47 per 100,000 for PV, ET, and PMF, respectively [4]. There was wide variation in incidence rates in the reviewed studies, which included global data from 1935 to 2010. Newer data from the Surveillance, Epidemiology and End Results (SEER) Program in the USA reported overall incidence rates from 2001 to 2016 to be 1.18, 1.14, and 0.33 per 100,000 for PV, ET, and PMF, respectively [5]. The Australian Cancer Registry started collecting MPN data in 2003 and reported incidence rates between then and 2014 to be 0.90, 0.95, and 0.45 per 100,000 for PV, ET, and PMF, respectively [6]. 

Internationally, differences in gender, age and ethnicity have been reported to affect the outcomes of MPN. Gender incidence rates are higher in males for PV and PMF, and higher in females for ET [4,5,6]. Incidence and mortality risk increase with age; the median age of diagnosis is in the 60–70 age range in nations with mostly European ethnic populations [5,6,7,8,9,10], and in the 50–60 age range in Asian nations [11,12,13,14]. The excess hazard was near 3.0 (95% CI: 2–4) for those aged 50–69 compared to those aged 15–49, and near 7.3 (6–10) in those aged 70–89 [5,6,7,8,9,10,11,12,13,14].

MPN have different incidence rates across ethnicities in the USA [8]. Whites have the highest incidence of PV (incidence rate (IR)—12.3) and PMF (IR—3.3) compared to Hispanics (PV IR—7.2; PMF IR—2.2), Blacks (PV IR—7.5; PMF IR—2.4), Asian and Pacific Islanders (PV IR—7.5; PMF IR—2.4). For ET, Blacks have the highest incidence (IR—11.5) compared to other ethnic groups [8]. Black patients diagnosed with PV (Hazard ratio (HR): 1.51; *p* = 0.004) and ET (HR: 1.63; *p* = 0.001) have a higher risk of death compared to White patients [15]. The authors suggested this may have been due to differential access to healthcare. In a single institution study where all patients had equal access to healthcare, Black patients with MPN were at higher risk of haemorrhagic complications than White patients, but there was no difference in overall survival [16]. 

Ethnic disparities have also been observed for PV in New Zealand [17]. In a single centre study, New Zealand (NZ) Polynesian patients were diagnosed with PV at a significantly younger age (mean 54 years) than Europeans (mean 68 years), but there was no difference in overall survival. Nevertheless, due to the younger age at diagnosis, NZ Polynesian patients had shorter life expectancies compared with the general population [17]. There are no other epidemiological or outcome data for MPN in New Zealand. 

The New Zealand Cancer Registry (NZCR) was established in 1984 and has been collecting data for PV and ET from 2010 and for PMF from 2014. The aim of this study was to review the demographics and outcomes of patients with PV, ET and PMF in New Zealand and, secondly, to describe the capture rates of these MPNs by the NZCR. The overriding aim was to assist further clinical research on MPN in New Zealand. Findings are expected to identify at risk populations and such epidemiological data may guide MPN screening recommendations in New Zealand and globally. 

## 2. Materials and Methods

### 2.1. Patient Cohort

This was a retrospective cohort study of a prospectively maintained national, population-based cancer registry. Patient data collected by the NZCR between 2010 and 2017 were analysed with a specific focus on PV, ET and PMF. MPNs were identified by International Classification of Diseases-10 (ICD-10) codes: D45 for PV, D47.3 for ET, and D47.4 for PMF. PMF was only recorded by the NZCR from 2014 when ICD-10-AM-VI was superseded by ICD-10-AM-VIII. Data were extracted for MPN patients of all ages on 14 June 2019 and deidentified; all patients recorded by the registry were analysed. Study procedures were approved by the Central Health and Disability Ethics Committee, approval number 16/CEN/92 granted on 8 August 2016.

### 2.2. The New Zealand Cancer Registry

The NZCR is a population-based register of all new malignant tumours diagnosed in New Zealand. The primary function of the registry is to accumulate information on the incidence and mortality from cancer to provide a basis for cancer research. The registry follows the guidelines for recording and reporting cancer incidence recommended by the International Agency for Research on Cancer (IARC) and the International Association of Cancer Registries (IACR). 

Historically, the registration of bone marrow malignancies to the NZCR depended on bone marrow pathology reports. At the start of the last decade a concern was growing among New Zealand haematologists that reporting and registering of MPN was suboptimal. With the wide availability of molecular testing performed on peripheral blood samples, a proportion of patients with suspected MPN did not undergo a bone marrow biopsy and, consequently, such patients were not reported to the NZCR. To address this issue, in 2016 the national Laboratory Haematology Working Group in collaboration with the NZCR produced New Zealand guidelines on reporting of haematological malignancies. Haematology services were encouraged to consider bone marrow examination for all suspected cases of MPN or identify alternative ways of reporting cases where bone marrow examination was not performed. For the latter group, many centres adopted direct reporting by the clinician making the diagnosis. The feedback on this initiative from the NZCR and haematology services has been encouraging, but a formal audit has never been performed.

### 2.3. Statistical Analysis

The fields extracted from the NZCR included dates of diagnosis, birth and death, gender, prioritised ethnicity, and the district health board at which the diagnosis was made for the MPN of interest. Ethnicity was assigned according to the Ministry of Health’s Ethnicity Data Protocols, where the respondent must identify their own ethnicity (self-identification). The primary outcome was patient mortality and secondary outcomes were NZCR capture rates.

Data parametricity was assessed via Shapiro–Wilk test. Parametric data were reported as mean (standard deviation, SD), and nonparametric data as median (interquartile range, IQR). Continuous variables were compared between diagnosis and ethnicity via one-way analysis of variance (ANOVA) when data were parametric, and Kruskal–Wallis test when nonparametric. Categorical variables were compared between diagnosis and ethnicity via Chi-square test. Kaplan–Meier plots were used to visualise and compare the survival between groups (defined by diagnosis, gender and ethnicity). Survival analysis of patients with variable follow-ups was conducted using a Cox proportional hazards regression model to produce hazard ratios (HRs) with 95% confidence intervals (CIs). The demographic factors, patient gender, age and ethnicity were introduced as predictors of mortality and were retained in all multivariate models as they are known confounders of outcomes in MPN. Age was analysed as a continuous variable by single year increments. The assumption of proportional hazards was assessed via log(-log) plots and Schoenfeld residuals [18]. If the assumption of proportional hazards was not met, time-varying variables were used by adding an interaction for time. Statistical analysis was performed using R version 3.6.1 software (R Foundation for Statistical Computing, Vienna, Austria). A *p* < 0.05 was considered significant.

## 3. Results

### 3.1. Capture Rates of PV, ET and PMF

**Figure 1:**
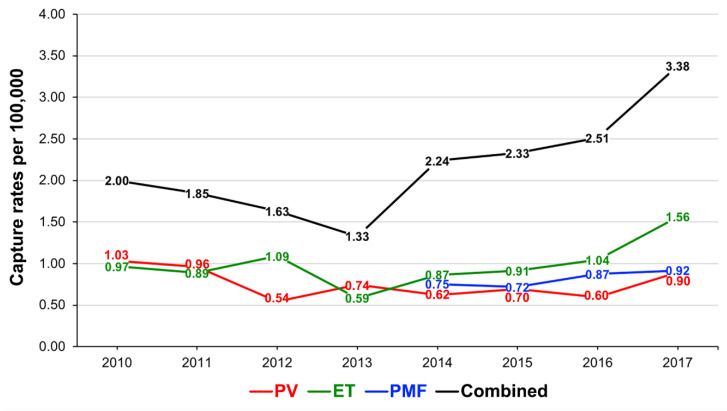
New Zealand Cancer Registry capture rates of classical Ph-negative myeloproliferative neoplasms (MPNs). Capture rates by the New Zealand Cancer Registry are shown per year for all MPNs, PV, ET and PMF, from 2010 to 2017. N.B. PMF registrations started in 2014. PV, polycythaemia vera; ET, essential thrombocythaemia; PMF, primary myelofibrosis.

Between 2010 and 2017, a total of 787 patients were reported to the NZCR and diagnosed with one of the three MPNs of interest; 275 (34.9%) had PV, 360 (45.7%) had ET, and 152 (19.3%) had PMF. PMF patients were only captured by the NZCR from 2014. Overall, prior to 2014, MPN capture rates were relatively modest, not exceeding 2.0 per 100,000 for all MPNs (Figure 1, Supplementary Table S1). The discovery of *CALR* mutations in 2013 led to an increase in MPN registrations in 2014, in particular for ET (Figure 1). Further increases occurred in 2017 after the introduction of national reporting guidelines in 2016 (as outlined in the Methods section). From 2016 to 2017, MPN capture rates increased by 50% for ET, 33% for PV and 6% for PMF. In 2017, the annual capture rate was the highest for ET (1.56 per 100,000), followed by PMF (0.92 per 100,000), and was the lowest for PV (0.90 per 100,000) (Figure 1, Supplementary Table S1). Most MPN cases were reported from the Auckland region (29.7%), which is the most populous in New Zealand, followed by the Canterbury region of the South Island (14.7%; Supplementary Table S2), roughly reflecting the population distribution in the country but no formal analysis into regional differences was performed. 

### 3.2. Overall Characteristics of Patients with MPN

Median age at diagnosis for all 787 MPN patients was 69.0 (IQR 58.0–78.0) years (Table 1). 

**Figure 2:**
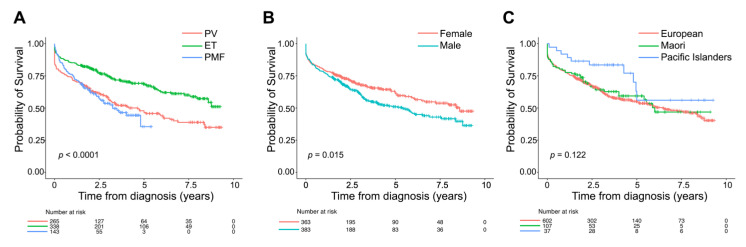
Survival of patients with Ph-negative myeloproliferative neoplasm reported to the New Zealand Cancer Registry between 2010 and 2017. Kaplan–Meier survival curves comparing cumulative survival probability of the entire cohort of myeloproliferative neoplasms patients stratified by diagnosis (**A**), gender (**B**), and main ethnic groups (**C**). Log-rank *p* values are shown. PV, polycythaemia vera; ET, essential thrombocythaemia; PMF, primary myelofibrosis.

Most MPN patients were identified as European (76.5%), with 18.3% being Polynesian (13.6% Māori and 4.7% Pacific Islanders), which is proportionate to the composition of the New Zealand population (Supplementary Table S3) [18]. Overall, by 14 June 2019, 318 (40.4%) patients died (Supplementary Table S4). ET patients had the best survival (log-rank; *p* < 0.001, Figure 2A, Supplementary Tables S4 and S5). Survival probability was similar in PV and PMF, which was unexpected and likely due to limited PMF registrations (4 years only) and late reporting of PV cases (see below). MPN patients who died were older when diagnosed (76.3 vs. 61.2 years, *p* < 0.001) and more often male (56.3%; *p* = 0.017, Supplementary Table S4; log-rank; *p* = 0.015, Figure 2B). There was no clear difference in the survival of Polynesian and European patients for all cases combined in unadjusted analyses (Figure 2C).

### 3.3. Epidemiology of Polycythaemia Vera

**Figure 3:**
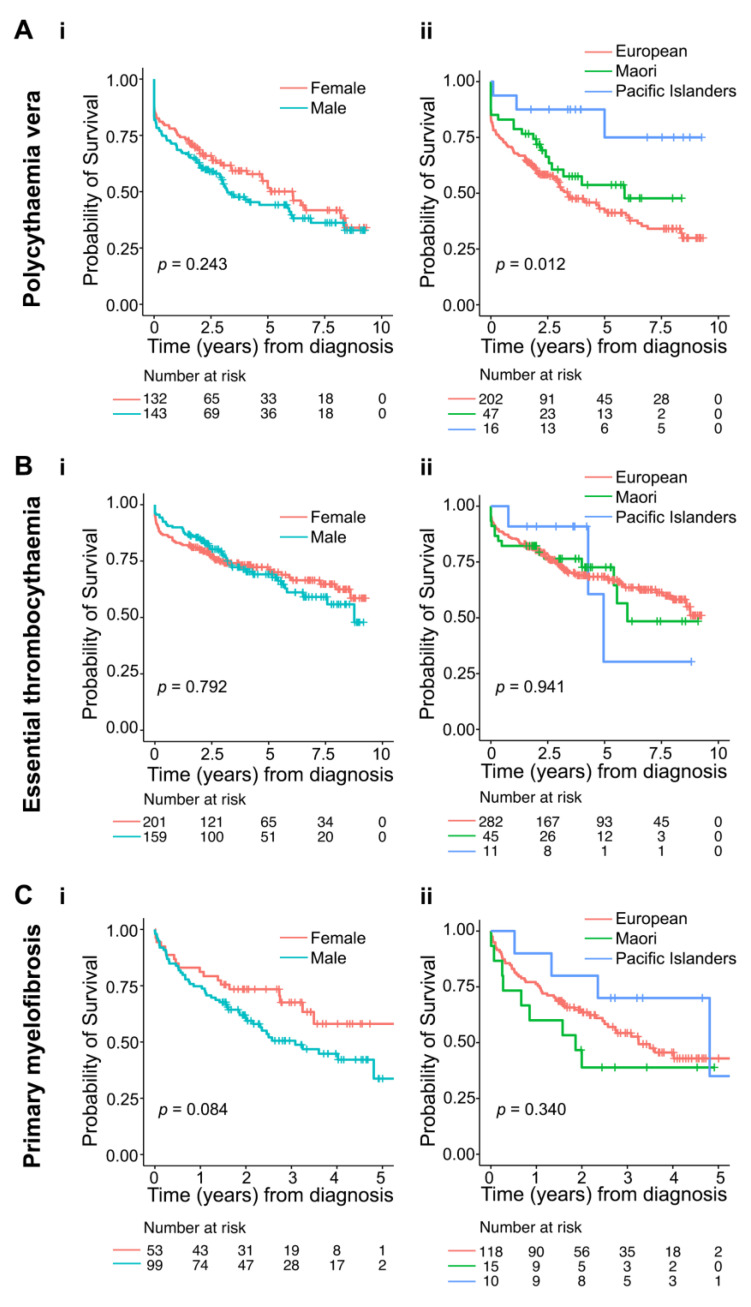
Influence of gender and ethnicity on survival of patients with myeloproliferative neoplasms. Kaplan–Meier survival curves for patients with (**A**) polycythaemia vera, (**B**) essential thrombocythaemia, and (**C**) primary myelofibrosis reported to the New Zealand Cancer Registry during 2010–2017, stratified by gender (**i**) and ethnicity (**ii**). Log-rank *p* values are shown.

Amongst 275 patients with PV, Māori and Pacific Island patients were significantly younger at diagnosis than European patients (59.5, 56.8 and 71.7 years, respectively; *p* < 0.001) (Table 1). The median length of follow-up for PV patients was 2.4 years (range 0–9.3). During this time, 51.3% patients died. The death rates were similar among Europeans (55.9%) and Māori (42.6%), but much lower amongst Pacific Islanders (18.9%, *p* < 0.001, Table 1; log-rank *p* = 0.012) (Figure 3A).

Both Māori and Pacific Island patients died at significantly younger ages than Europeans (68.9, 66.1 and 81.0 years, respectively; *p* = 0.001; Table 1). After adjusting for age, gender and ethnicity, increasing age and male gender were identified as independent risk factors for mortality in PV. Mortality risk increased 7% with each year of age (HR: 1.07, 95% CI: 1.05–1.1%; *p* = 0.001; Table 2). Males had a 43% higher risk of dying than females (HR: 1.43 95% CI: 1.01–2.00; *p* = 0.04; Table 2).

### 3.4. Epidemiology of Essential Thrombocythaemia

Amongst 360 patients with ET, Māori and Pacific Island patients were significantly younger at diagnosis than their European counterparts (60.5, 61.1 and 67.4 years, respectively; *p* = 0.004; Table 1). The median length of follow-up for ET was 3.2 years (range 0–9.3). During this period, 30.3% patients died. The death rates were similar across ethnicities (*p* = 0.310, Table 1, Figure 3B). Univariate analysis showed no demographic factors influencing mortality (Table 2). Similarly, after adjusting for age, gender, ethnicity, and follow-up time, we found no significant independent risk factors for mortality in ET (Table 2).

### 3.5. Epidemiology of Primary Myelofibrosis

Similar to PV and ET, Māori and Pacific Island patients with PMF were younger at diagnosis than European patients (61.5, 65.3 and 71.6 years, respectively; *p* < 0.001; Table 1). 

The median length of follow-up for the 152 patients with PMF was 2.0 years (range 0–5.4), which was shorter compared to PV and ET due to a later starting of NZCR registrations. The death rate amongst all reported PMF patients was 44.7% over the median follow-up time of 2 years. After adjusting for age, gender and ethnicity (Table 2), age, male gender and Māori ethnicity were identified as independent risk factors for mortality. There was a 6% increase per year (HR: 1.06, 95% CI: 1.04–1.1; *p* < 0.001) in mortality risk with increasing age. Males were 81% (HR: 1.81, 95% CI: 1.05–3.1; *p* = 0.034) more likely to die than females. Māori ethnicity tripled (HR: 2.94, 95% CI: 1.37–6.3; *p* = 0.006) the risk of mortality compared to Europeans (Table 2, Figure 3C).

## 4. Discussion

This study investigated the incidence and mortality outcomes of patients with classical Ph-negative MPN (PV, ET and PMF) in New Zealand based on the data reported to the NZCR between 2010 and 2017 for PV and ET, and between 2014 and 2017 for PMF. We found that capture rates of MPN increased in 2014 and peaked in 2017, reaching annual incidence rates of 3.38, 0.90, 1.56 and 0.92 per 100,000 for all MPNs, PV, ET, and PMF, respectively. Māori and Pacific Island patients presented at younger ages in PV, ET and PMF and died earlier in PV and PMF. Male gender was a risk factor for mortality in PV and PMF, with nearly double the risk of mortality. Māori ethnicity nearly tripled the risk of mortality in PMF. There were no significant demographic risk factors for mortality identified for patients with ET. Our study provides important and novel epidemiological findings not previously known about MPNs in New Zealand. 

In 2016, the NZCR reporting guidelines were introduced and in effect, MPN registrations peaked in 2017. The biggest increase was for PV (from 0.60 per 100,000 in 2016 to 0.90 in 2017), suggesting that prior to 2017, many PV cases were not reported to the NZCR if diagnosed solely on the peripheral blood and molecular criteria. Overall, average MPN capture rates in New Zealand between 2010 and 2017 (0.76, 0.99, and 0.82 per 100,000 for PV, ET, and PMF, respectively) are within previously reported international incidence ranges of 0.01–2.61, 0.21–2.27, and 0.22–0.99 per 100,000 for PV, ET, and PMF, respectively [4,5]. However, they are lower than the average international rates for PV and ET (0.84 and 1.03 per 100,000, respectively), but higher than the average international incidence rate for PMF (0.47 per 100,000) [4,19]. PMF rates captured by the NZCR are of the highest in the world (0.82 per 100,000 compared with 0.3–0.4 from 2001 to 2016 in the USA and ~0.45 between 2003 and 2014 in Australia) [5,6]. It is possible that some patients had secondary myelofibrosis (MF), either post-PV or -ET. It can be challenging to diagnostically differentiate secondary from primary MF, as well as ET from early PMF [20]. If MF was diagnosed late in disease progression, an antecedent PV or ET may not have been captured. Underreporting and late reporting may have introduced bias; follow-up analyses of the NZCR data should help clarify our findings.

The median age of MPN patients diagnosed in nations with majority European ethnic populations is generally in the 60s to early 70s, with the youngest being for ET and oldest for PMF [5,6,7,8,9,10,21,22]. This was similar in our study—mean age at diagnosis was 65.5 years for ET patients and 69.2 years for PMF patients. Gender differences between NZCR and international data were also similar, showing higher incidence among females for ET and males for PV and PMF [4,5,6]. 

Due to short-term follow-up, our mortality rates are difficult to compare to USA cohorts, but there are similarities. PMF is known to be associated with younger age of death, and ET has good survival rates [1,5,8,9]. This is reflected in the best outcomes and older age at death in ET (mean 80.2 years), and younger age at death in PMF (mean 74.8 years) in our cohort. However, the comparable survival between PMF and PV in our study is unexpected. This is likely due to late reporting of PV cases (suggested by their relatively older age and low PV capture rates), further confounded by the short time period of PMF records available (2014–2017). Despite these limitations, we identified male gender as an independent risk factor for mortality in PV and PMF, consistent with other reports [23,24,25]. Recent study demonstrated that men have a higher mutational burden in CD34-positive cells and higher risk of non-MPN-specific high-risk mutations including in *ASXL1*, *EZH2*, *SRSF2*, *U2AF1*, and *IDH1/2* genes compared with women [25]. It would be interesting to test for such mutations in New Zealand patients in the future.

Our work uncovered some intriguing ethnic differences in demographic characteristics of MPN in New Zealand. For PV, Māori and Pacific Islanders were younger at diagnosis than Europeans (median age of 63.0, 56.0 and 73.0 years, respectively). Māori and Pacific Islander patients also died younger than European patients (median age of 68.7, 66.1 and 81.0 years, respectively). We have previously reported that NZ Polynesians with PV treated at Middlemore Hospital (Auckland) presented at a significantly younger age than Europeans [17], and that finding has been replicated in this study. 

For ET, the median age of diagnosis in Māori and Pacific Islanders was also significantly younger than Europeans, but not as pronounced as for PV patients. However, the median age of death from ET was not significantly different between the ethnic groups, suggesting the natural history of ET is similar between ethnicities. 

The median age of diagnosis of PMF for Māori and Pacific Islanders was significantly lower than Europeans (57.0, 62.5 and 71.0 years, respectively). Māori PMF patients also died at a younger median age than Pacific Island and European PMF patients (60.3, 75.9 and 75.4 years, respectively). The percentage of Māori PMF patients that died was higher than Pacific Island and European PMF patients (60.0%, 40.0% and 46.6%, respectively). There was an unusually high percentage (80%) of Māori and Pacific Islanders males with PMF, but neither of these percentages reached significance. After adjusting for age, gender and ethnicity, Māori ethnicity tripled the risk of PMF mortality compared to Europeans (HR: 2.94, 95% CI: 1.37–6.3; *p* = 0.006).

The Dynamic International Prognostic Scoring System-Plus (DIPSS-Plus) uses a set of risk factors to predict survival in PMF [26]. It is unknown whether Māori ethnicity increases the likelihood of having these risk factors, and further studies are required. Cardiovascular complications are a common cause of death in PMF [27]. Māori have higher rates of cardiovascular disease than Europeans and Pacific Islanders [28], which may have contributed to higher death rates. Māori are also overrepresented in areas of higher deprivation; thus societal inequities in access to healthcare may have affected worse outcomes [29]. 

Our previous research found that NZ Polynesian patients with PV [17] and acute promyelocytic leukaemia [30] also present at a younger age. Together, this raises a possibility that NZ Polynesians have a unique set of risk factors or a form of genetic predisposition to myeloid neoplasia. Polynesians smoke more tobacco and have higher rates of obesity, which increases the risk of certain cancers [31,32,33], and is of relevance in MPN [34,35,36,37,38,39]. Risk factors for MPN remain poorly defined; however, certain modifiable lifestyle factors have been implicated (e.g., diet, smoking, alcohol and coffee consumption, psychological stress and physical activity), as well as certain chemical exposures (e.g., benzene and polycyclic aromatic hydrocarbons) [39,40]. Better definition of the roles of these risk factors in MPN pathogenesis may help design new preventative strategies against MPN. Future studies in New Zealand should therefore record lifestyle and environmental risk factors in the MPN cohorts to determine how these factors impact MPN presentation and outcomes in Polynesian and European patients. Evaluation of associated inflammation would also be of interest, in particular in the bone marrow microenvironment [41]. 

Inherited factors play a role in the development and biology of MPN [42,43]. Research into genomic associations between ethnicity and MPN incidence and outcomes in the New Zealand population is important to undertake. Similar genetic associations, for example the E-Cadherin (*CDH1*) gene mutations of hereditary diffuse gastric cancer and its familial associations with Māori ethnicity, have had a revolutionary clinical impact in the New Zealand setting [44]. We advocate for genome-wide association studies to be performed in NZ Polynesian and European patients with and without MPN to seek an underlying cause. Results may ultimately lead to novel preventative, diagnostic and therapeutic measures for NZ Polynesians and other patients. 

More research into ethnic differences in haematological cancers is needed globally. Further advances in clinical outcomes will require an understanding of the drivers of ethnicity-based differences in cancer outcomes. A better understanding of these drivers will allow prioritisation of research and clinical funding to serve populations with more need [45,46]. Future discoveries in this area have the potential to improve both patient and public health outcomes. The results may have implications for recommending increased screening of extended families of MPN patients.

Our study should be considered in light of its limitations. PMF data were available only from 2014 to 2017, covariates were limited, and the follow-up time was short compared to other larger cohorts [5,19,47]. Many confounders such as comorbidities and patient factors influence patient mortality; however, these were not available for analysis and should be considered in the design of future clinical studies. Clinicopathological variables, in particular indicators of disease severity, would be important confounders to adjust for in future analyses of MPN mortality. Other studies have adjusted for laboratory variables such as white blood cell counts, cytogenetics and molecular results, splenomegaly, thrombotic events, major bleeding events, and transplant status [16]. The reason why risk factors for ET mortality were not identified is unclear but some of the confounding factors above and inadequate reporting may have contributed. The NZCR may not yet comprehensively captured the true incidence of reported MPNs, but this is a continually improving process. Further reviews of the NZCR data will be undertaken in the future to help clarify true incidence trends and risk factors for MPN in New Zealand. 

A strength of this study is its relatively large population size for each MPN subgroup. As this cohort includes patients from all over New Zealand, results are likely generalisable to other populations and provide insights about MPN that may be useful in an international context. Ruxolitinib, prescribed to improve MPN signs and symptoms, has only been funded in New Zealand since October 2018. Therefore, the NZCR outcome data are not impacted by the use of this drug. As novel therapies are introduced moving forward, NZCR provides an invaluable source of unbiased information to monitor impact of new treatments on patient survival. Our results highlight groups of MPN patients that are at high risk of mortality in New Zealand that should be reached with novel therapies—these are PMF patients, in particular Māori and male patients. Baring limitation of biased reporting, PV patients also did not fare well, highlighting the need for novel treatments for high-risk PV patients.

## 5. Conclusions

In conclusion, the NZCR is a reliable haematologic cancer registry consistent with international registries. It is a valuable tool for monitoring the incidence and mortality of MPN in New Zealand. We identified that increasing age and male gender are independent risk factors of mortality in PV and PMF and notably, Māori ethnicity was associated with a three times higher mortality risk in PMF compared to European ethnicity. Further work is required to identify modifiable risk factors for mortality in MPN. It is important that future therapeutic trials in New Zealand target high-risk patients identified in this study and that genetic research is undertaken to examine the underlying cause. 

## Figures and Tables

**Table 1:** Characteristics of three classical Ph-negative myeloproliferative neoplasms captured by the New Zealand Cancer Registry, 2010–2017 (*n* = 787).

**Polycythaemia Vera**					
	Total *	**European**	**Māori**	**Pacific Islanders**	***p* Value**
N (%)	275 (100%)	202 (73.5%)	47 (17.1%)	16 (5.8%)	
Age at diagnosis, mean (SD)	68.6 (15.0)	71.7 (13.9)	59.5 (15.8)	56.8 (12.3)	<0.001
Age at diagnosis, median (IQR)	68.0 (55.0, 77.0)	73.0 (64.0, 82.0)	63.0 (48.5, 69.0)	56.0 (48.8, 62.0)	
Gender (%)					
Female	132 (48.0)	98 (48.5)	21 (44.7)	6 (37.5)	0.481
Male	143 (52.0)	104 (51.5)	26 (55.3)	10 (62.5)	
Number of patients that died (%)	141 (51.3)	113 (55.9)	20 (42.6)	3 (18.9)	0.017
Age at death, median (IQR)	81.0 (74.4, 87.2)	81.0 (73.1, 87.4)	68.7 (63.2, 77.0)	66.1 (60.1, 77.1)	0.001
**Essential Thrombocythaemia**					
	**Total**	**European**	**Māori**	**Pacific Islanders**	***p* Value**
N (%)	360 (100%)	282 (78.3%)	45 (12.5%)	11 (3.1%)	
Age at diagnosis, mean (SD)	65.5 (16.0)	67.4 (15.3)	60.5 (16.9)	61.1 (16.6)	0.004
Age at diagnosis, median (IQR)	69.5 (60.0, 78.0)	70.0 (58.0, 78.0)	62.0 (52.0, 70.0)	64.0 (46.5, 76.0)	
Gender (%)					
Female	201 (55.8)	154 (54.6)	27 (60.0)	7 (63.6)	0.818
Male	159 (44.2)	128 (45.4)	18 (40.0)	4 (36.4)	
Number of patients that died (%)	109 (30.3)	91 (32.3)	14 (31.1)	3 (27.3)	0.310
**Age at death, median (IQR)**	74.6 (66.4, 84.7)	81.6 (75.3, 87.7)	78.7 (64.3, 84.4)	81.0 (78.9, 81.1)	0.379
**Primary Myelofibrosis**					
	**Total**	**European**	**Māori**	**Pacific Islanders**	***p* Value**
N (%)	152 (100%)	118 (77.6%)	15 (9.9%)	10 (6.6%)	
Age at diagnosis, mean (SD)	69.2 (12.7)	71.6 (10.8)	61.5 (18.4)	65.3 (8.1)	<0.001
Age at diagnosis, median (IQR)	70.0 (59.0, 80.0)	71.0 (65.0, 80.0)	57.0 (53.0, 66.0)	62.5 (59.3, 72.3)	
Gender (%)					
Female	53 (34.9)	45 (38.1)	3 (20.0)	2 (20.0)	0.369
Male	99 (65.1)	73 (61.9)	12 (80.0)	8 (80.0)	
Number of patients that died (%)	68 (44.7)	55 (46.6)	9 (60.0)	4 (40.0)	0.063
Age at death, median (IQR)	79 (71.0, 86.0)	75.39 (68.1, 84.8)	60.3 (55.6, 92.0)	75.9 (71.2, 78.7)	0.443

SD, standard deviation; IQR; interquartile range; N, number. * This column includes patients of all ethnicities; further details are shown in Supplementary Tables S3 and S4.

**Table 2:** Cox-regression model of predictors of mortality in MPN patients captured by the New Zealand Cancer Registry during 2010–2017.

	**Univariate Analysis**			**Multivariate Analysis**		
**Polycythaemia vera**						
	**HR for Death**	**95% CI**	***p* Value**	**HR for Death**	**95% CI**	***p* Value**
Age	1.07	1.05–1.09	<0.001	1.07	1.05–1.1	0.001
Gender						
Female	Ref			Ref		
Male	1.26	0.90–1.77	0.182	1.43	1.01–2.0	0.041
Ethnicity *						
European	Ref			Ref		
Māori	0.71	0.44–1.15	0.165	1.46	0.89–2.4	0.136
Pacific Islanders	0.23	0.07–0.73	0.013	0.49	0.15–1.6	0.231
**Essential thrombocythaemia**						
	**HR for Death**	**95% CI**	***p* Value**	**HR for Death**	**95% CI**	***p* Value**
Age	1.11	1.09–0.14	1.113	1.007	0.981–1.034	0.591
Gender ^#^						
Female	Ref		Ref	Ref		
Male	1.11	0.58–2.12	1.105	1.196	0.623–2.294	0.590
Ethnicity *^,#^						
European	Ref		Ref	Ref		
Māori	0.92	0.39–2.18	0.923	0.944	0.401–2.224	0.895
Pacific Islanders	0.84	0.11–6.43	0.842	1.320	0.182–9.561	
**Primary myelofibrosis**						
	**HR for Death**	**95% CI**	***p* Value**	**HR for Death**	**95% CI**	***p* Value**
Age	1.05	1.03–1.08	<0.001	1.06	1.04–1.1	<0.001
Gender						
Female	Ref			Ref		
Male	1.65	0.96–2.84	0.068	1.81	1.05–3.1	0.034
Ethnicity *						
European	Ref					
Māori	1.45	0.72–2.95	0.300	2.94	1.37–6.3	0.006
Pacific Islanders	0.63	0.23–1.74	0.371	0.75	0.26–2.1	0.592

* This analysis excluded patients with ethnicities other than European, Māori and Pacific Islanders. ^#^ Both gender and ethnicity did not meet the assumption of proportional hazards, hence univariate hazard ratios (HRs) were adjusted for an interaction with time in both cases. Multivariate models were adjusted for an interaction between gender and time and also ethnicity and time to account for the lack of proportional hazards. CI, confidence interval.

## Data Availability

Datasets generated for the current study are not publicly available considering confidentiality reasons. Deidentified data may be available from the corresponding author on justified request.

## Supplementary Materials

The following are available online at https://www.mdpi.com/article/10.3390/curroncol28020146/s1, Table S1: The New Zealand Cancer Registry capture rates of patients with myeloproliferative neoplasms during 2010–2017, Table S2: Numbers of patients with myeloproliferative neoplasms reported to the New Zealand Cancer Registry from different New Zealand District Health Boards, Table S3: Demographic factors of patients with myeloproliferative neoplasms reported to the New Zealand Cancer Registry, Table S4: Comparison of baseline demographics for patients with myeloproliferative neoplasms according to their survival status, Table S5: The New Zealand Cancer Registry death rates for patients with myeloproliferative neoplasms.
