# Supplementary material for: The Epidemiology of Myeloproliferative Neoplasms in New Zealand between 2010 and 2017: Insights from the New Zealand Cancer Registry
Source: Curr Oncol. 2021 Apr 18;28(2):1544–57. doi: 10.3390/curroncol28020146 (PMC8167767; doi:10.3390/curroncol28020146)
Supplement: Supplementary file 1 [file curroncol-28-00146-s001.zip › curroncol-1153080-supplementary.pdf]

Supplementary Material

The Epidemiology of Myeloproliferative Neoplasms in New Zealand between 2010 and 2017: Insights from the New Zealand Cancer Registry

Chris Varghese, Tracey Immanuel, Anna Ruskova, Edward Theakston and Maggie L. Kalev-Zylinska

Table S1. The New Zealand Cancer Registry capture rates of patients with myeloproliferative neoplasms during 2010-2017.

Numbers of patients registered per year	2010	2011	2012	2013	2014	2015	2016	2017	Aver-age
**All**	87	81	72	59	101	107	118	162	98
**PV**	45	42	24	33	28	32	28	43	34
**ET**	42	39	48	26	39	42	49	75	45
**PMF**	-	-	-	-	34	33	41	44	38
**New Zealand population**	4,351,000	4,384,000	4,408,000	4,442,000	4,510,000	4,596,000	4,693,000	4,794,000	
**Overall capture per 100,000**	2	1.85	1.63	1.33	2.24	2.33	2.51	3.38	2.16
**PV capture per 100,000**	1.03	0.96	0.54	0.74	0.62	0.70	0.60	0.90	0.76
**ET capture per 100,000**	0.97	0.89	1.09	0.59	0.87	0.91	1.04	1.56	0.99
**PMF capture per 100,000**	-	-	-	-	0.75	0.72	0.87	0.92	0.82

Table S2. Numbers of patients with myeloproliferative neoplasms reported to the New Zealand Cancer Registry from different New Zealand District Health Boards.

		Overall	%	PV	%	ET	%	PMF	%
**N**		787		275		360		152	
**DHB**	**Canterbury**	116	14.7	37	13.5	57	15.8	22	14.5
	**Counties Manu-kau**	88	11.2	26	9.5	41	11.4	21	13.8
	**Auckland**	78	9.9	35	12.7	34	9.4	9	5.9
	**Waitemata**	68	8.6	21	7.6	33	9.2	14	9.2
	**Southern**	61	7.8	24	8.7	30	8.3	7	4.6
	**Waikato**	60	7.6	16	5.8	27	7.5	17	11.2
	**MidCentral**	54	6.9	20	7.3	25	6.9	9	5.9
	**Capital and Coast**	46	5.8	21	7.6	13	3.6	12	7.9
	**Hawkes Bay**	37	4.7	11	4.0	22	6.1	4	2.6
	**Northland**	27	3.4	7	2.5	19	5.3	1	0.7
	**Taranaki**	27	3.4	14	5.1	12	3.3	1	0.7
	**Bay of Plenty**	26	3.3	5	1.8	11	3.1	10	6.6
	**Nelson Marlbor-ough**	20	2.5	13	4.7	3	0.8	4	2.6
	**South Canterbury**	17	2.2	5	1.8	6	1.7	6	3.9
	**Hutt Valley**	16	2.0	7	2.5	6	1.7	3	2.0
	**Lakes**	16	2.0	5	1.8	6	1.7	5	3.3
	**Other**	16	2.0	4	1.5	5	1.4	7	4.6
	**Whanganui**	14	1.8	4	1.5	10	2.8	0	0.0

DHB, District Health Board.

Table S3. Demographic factors of patients with myeloproliferative neoplasms reported to the New Zealand Cancer Registry.

	Overall	Polycythaemia Vera	Essential Thrombocythaemia	Primary Myelofibrosis*	*P* value
**N**	787	275 (34.9%)	360 (45.7%)	152 (19.3%)	
**Age at diagnosis, mean (SD)**	67.3 (15.2)	68.6 (15.0)	65.5 (16.0)	69.2 (12.7)	**0.008**
**Age at diagnosis, median (IQR)**	70.0 (59.0, 80.0)	68.0 (55.0, 77.0)	69.5 (60.0, 78.0)	70.0 (59.0, 80.0)	
**Gender (%)**					
Female	386 (49.0)	132 (48)	201 (55.8)	53 (34.9)	**<0.001**
Male	401 (51.0)	143 (52)	159 (44.2)	99 (65.1)	
**Ethnicity (%)**					
European	602 (76.5)	202 (73.5)	282 (78.3)	118 (77.6)	0.149
Maori	107 (13.6)	47 (17.1)	45 (12.5)	15 (9.9)	
Pacific Islanders	37 (4.7)	16 (5.8)	11 (3.1)	10 (6.6)	
Asian	27 (3.4)	7 (2.5)	13 (3.6)	7 (4.6)	
Other	14 (1.8)	3 (1.1)	9 (2.5)	2 (1.3)	
**Numbers of patients that died (%)**	318 (40.4)	141 (51.3)	109 (30.3)	68 (44.7)	**0.001**
**Age at death, mean (SD)**	77.9 (11.1)	77.7 (11.2)	80.2 (9.8)	74.8 (12.0)	**0.007**
**Age at death, median (IQR)**	79.0 (71.0, 86.0)	81.0 (74.4, 87.2)	74.6 (66.4, 84.7)	79.0 (71.0, 86.0)	

*PMF diagnosis includes patients from different time periods.

Table S4. Comparison of baseline demographics for patients with myeloproliferative neoplasms according to their survival status.

	Overall	Alive*	Dead*	*P* value
**N (%)**	787	469 (59.6%)	318 (40.4%)	
**Age at diagnosis, mean (SD)**	67.3 (15.2)	61.2 (14.4)	76.3 (11.3)	**<0.001**
**Age at diagnosis, median (IQR)**	70.0 (59.0, 80.0)	63.0 (53.0, 72.0)	77.0 (69.0, 84.0)	
**Age at death, mean (SD)**	-	-	77.9 (11.1)	
**Age at death, median (IQR)**	-	-	79.0 (71.5, 86.5)	
**MPN type (%)**				
Polycythaemia vera	275	134 (48.7)	141 (51.3)	**<0.001**
Essential thrombocythaemia	360	251 (69.7)	109 (30.3)	
Primary myelofibrosis	152	84 (55.3)	68 (44.7)	
**Gender (%)**				
Female	386	247 (64.0)	139 (36.0)	**0.017**
Male	401	222 (55.4)	179 (44.6)	
**Ethnicity (%)**				
European	602	343 (73.1)	259 (81.5)	**0.002**
Maori	107	64 (13.6)	43 (13.5)	
Pacific Islanders	37	27 (5.8)	10 (3.1)	
Asian	27	23 (4.9)	4 (1.3)	
Other	14	12 (2.6)	2 (0.6)	

* As of 14 June 2019.

Table S5. The New Zealand Cancer Registry death rates for patients with myeloproliferative neoplasms.

Numbers of patients that died per year	2010	2011	2012	2013	2014	2015	2016	2017	Aver-age
**All**	48	37	38	35	59	34	39	28	40
**PV**	30	24	18	22	20	14	8	5	18
**ET**	18	13	20	13	18	5	11	11	14
**PMF**	-	-	-	-	21	15	20	12	17
**New Zealand population**	4,351,000	4,384,000	4,408,000	4,442,000	4,510,000	4,596,000	4,693,000	4,794,000	
**Overall deaths per 100,000**	1.10	0.84	0.86	0.79	1.31	0.74	0.83	0.58	0.88
**PV deaths per 100,000**	0.69	0.55	0.41	0.50	0.44	0.31	0.17	0.10	0.40
**ET deaths per 100,000**	0.41	0.30	0.45	0.29	0.40	0.11	0.23	0.23	0.30
**PMF deaths per 100,000**	-	-	-	-	0.47	0.33	0.43	0.25	0.37
